# Feasibility study of assessing the Preclinical Alzheimer Cognitive Composite (PACC) score via videoconferencing

**DOI:** 10.1007/s00415-021-10403-1

**Published:** 2021-01-26

**Authors:** Giulia Seghezzo, Yvonne Van Hoecke, Laura James, Donna Davoren, Elizabeth Williamson, Neil Pearce, Damien McElvenny, Valentina Gallo

**Affiliations:** 1 Institute of Population Health Sciences, Queen Mary University of London, London, UK; 2 London School of Hygiene and Tropical Medicine, London, UK; 3 Institute of Occupational Medicine, Edinburgh, UK; 4 Centre for Occupational and Environmental Health, University of Manchester, Manchester, UK; 5 Campus Fryslân, University of Groningen, Leeuwarden, The Netherlands

**Keywords:** Telemedicine, Cognitive testing, Cognitive decline, Mild cognitive impairment

## Abstract

**Background:**

The Preclinical Alzheimer Cognitive Composite (PACC) is a composite score which can detect the first signs of cognitive impairment, which can be of importance for research and clinical practice. It is designed to be administered in person; however, in-person assessments are costly, and are difficult during the current COVID-19 pandemic.

**Objective:**

To assess the feasibility of performing the PACC assessment with videoconferencing, and to compare the validity of this remote PACC with the in-person PACC obtained previously.

**Methods:**

Participants from the HEalth and Ageing Data IN the Game of football (HEADING) Study who had already undergone an in-person assessment were re-contacted and re-assessed remotely. The correlation between the two PACC scores was estimated. The difference between the two PACC scores was calculated and used in multiple linear regression to assess which variables were associated with a difference in PACC scores.

**Findings:**

Of the 43 participants who were invited to this external study, 28 were re-assessed. The median duration in days between the in-person and the remote assessments was 236.5 days (7.9 months) (IQR 62.5). There was a strong positive correlation between the two assessments for the PACC score, with a Pearson correlation coefficient of 0·82 (95% CI 0·66, 0·98). The multiple linear regression found that the only predictor of the PACC difference was the time between assessments.

**Interpretation:**

This study provides evidence on the feasibility of performing cognitive tests online, with the PACC tests being successfully administered through videoconferencing. This is relevant, especially during times when face-to-face assessments cannot be performed.

**Supplementary Information:**

The online version contains supplementary material available at 10.1007/s00415-021-10403-1.

## Introduction

Dementia is a growing public health challenge, with an estimated 40–50 million people living with this condition globally [1]. Worldwide, the prevalence of dementia more than doubled from 1990 to 2016, mainly due to the ageing population; it is now the 5th leading cause of death globally [1, 2]. Dementia onset is usually preceded by mild cognitive impairment (MCI), with population-based studies finding up to 22% of people with MCI developing dementia [1]. Currently, there is an increasing interest in the early diagnosis of dementia, to allow potential screening programs, as well as clinical trials testing disease-modifying drugs early on in the neuropathological process [3]. In this context, assessing patients at very early stages of MCI is important.

The Preclinical Alzheimer Cognitive Composite (PACC) is a composite score which combines tests that assess episodic memory, timed executive function, and global cognition, and it has been shown to be able to detect the first signs of cognitive decline, before clinical signs of MCI manifest [4]. The PACC score is increasingly used in epidemiological studies to assess an association between exposures and early changes in cognitive function [5, 6]. The PACC is designed to be administered in person, by a trained research psychologist or nurse. However, in epidemiological studies, in-person assessments are costly, often require extensive travelling, and are difficult in the current pandemic situation. Assessing cognitive function in older adults may be possible via videoconferencing, but there have been calls for further validation studies [7]. Some early studies have shown that remote video assessments are feasible on cognitively normal participants, as well as those with Alzheimer’s disease, dementia and Parkinson’s disease [8–12]. However, no study has assessed the feasibility of performing videoconference assessments in the participant’s home with their own equipment, with the majority of studies assessing feasibility by performing video assessments in clinics [7, 8, 11–13].

The HEalth and Ageing Data IN the Game of football (HEADING) Study is an ongoing study assessing the relationship between concussions and repetitive sub-concussive head injuries in retired football players, and cognitive function as measured with the PACC score. In March 2020, due to the COVID-19 pandemic, and the imposed lockdown by the UK government, the HEADING Study could no longer assess its participants in-person, prompting a need to find other modes of assessment. The aim of this study is to assess the feasibility of performing the PACC score via videoconferencing and comparing the validity of the remote PACC score with the in-person PACC score obtained previously, by recalling participants of the HEADING study who had already been assessed for a new remote assessment.

## Methods

### Source population

Participants in the HEADING Study were selected from the Professional Footballer’s Association (PFA) member database, a union for current and former professional football players of the English Premier League. Any male member over the age of 50 with an address in England was sent an invitation in the mail regarding the study, and a request to contact the study team to schedule an appointment. Appointments were held in clinics in London or Manchester, or at the participant’s home. The in-person assessment included a lifestyle questionnaire, exposure assessment questionnaire, and cognitive tests, in addition to some physical measures, the assessment protocol is similar to that of the BRAIN Study [5], apart from the addition of repetitive sub-concussive head injuries to the exposure assessment.

The HEADING Study recruitment was ongoing when, on 23/03/2020 due to the COVID-19 pandemic, a lockdown in the UK was announced and in-person assessment were no longer possible.

All participants who had already completed the in-person assessment for the HEADING Study between July 2019 and March 2020 were contacted by telephone and/or by email, requesting their voluntary participation in an additional remote assessment. Participants were asked if they had the capability to perform video calls, by having access either a computer, tablet or smartphone with a camera. Step-by-step instructions and over the phone support were offered to participants if they were not familiar with downloading or using any videoconferencing software (instructions provided for Skype and Zoom). If the participant agreed and met the requirements of joining a video call, an appointment was scheduled with a video-conferencing software the participant was most familiar with (Zoom^®^, Skype^®^, Microsoft Teams^®^, Facebook Portal^®^ or FaceTime^®^).

The HEADING Study was approved by the London School of Hygiene and Tropical Medicine’s Ethical Committee (16282). Participants were not involved in the design of the study nor of the present sub-study.

### In person assessment

The PACC score used in the HEADING Study is based on that used in the British 1946 Birth Cohort [6, 14] and in the BRAIN Study [5], and consists of the following:

- The Mini Mental State Examination (MMSE) total score (0–30 points): used to assess multiple cognitive domains including orientation to time and place, attention and calculation, recall, language, writing, visuospatial function, and executive function.
- The total score of the 12-item Face-Name Associative Memory Test (F-NAME 12A) (0–96 points): used to assess the ability of the participant to recall names and occupation of a number of people showed in pictures.
- The delayed recall score on the logical memory IIa subtest from the Weschler Memory Scale (0–25 story units): used to assess the ability to freely recall a short story.
- The Digit Symbol Substitution Test (DSST) score from the Weschler Adult Intelligence Scale Revised (0–93 symbols): used to assess attention and psychomotor speed.

Each of the four component scores was divided by the standard deviation (SD) of that component to form standardised *z*-scores. The mean of these *z*-scores was then calculated to form the composite score [4]. A complete PACC score for this study was defined as of having the MMSE and at least two other tests completed [5].

### Remote video assessments

Prior to the assessment participant packs were posted to the participant address which contained all materials necessary for their assessments. These included: (1) cover letter; (2) blank paper (for MMSE commands involving grabbing paper with right hand, folding paper and placing it on lap) (3) MMSE worksheets (draw pentagons and write sentence); (4) DSST worksheet; (5) Post Assessment Interview; (6) stamped return envelope.

As the DSST was a timed task, the worksheet was enclosed in a sealed envelope within the participant pack, with the following sentence ‘Please do not open until you are told to.’ Participants then opened the sealed envelope when instructed to do so by the research assistant during the remote assessment. Additional material available to each of the remote assessors included: (1) timer for the MMSE, F-NAME, Logical memory test, and DSST; (2) wristwatch for the MMSE; (3) Stimulus card for the MMSE; (4) PowerPoint file for the F-NAME; (5) hard copy of the narrative of the Logical Memory test; (6) hard copy of the worksheet for the DSST; and (7) pen for scoring the tests and taking notes.

The order of the tests was changed slightly from the order of the in-person assessment to fit with time restrictions required for the tests, as well as to ensure that the remote assessment was short and did not include too many gaps between tests (Box 1): there was a 20-min delay between the Immediate Recall Logical Memory Test and the Delayed Recall Logical Memory Test; similarly, there was a 30-min delay between the Cued Face Name Associative Memory Test and the Delayed Face Name Associative Memory Test. The tests were scheduled to take 60 min in total.

**Box 1:** Comparison of the order of the single components of the PACC when administered in-person and when administered remotely

Order of the tests comprising the PACC during the in-person assessment	Order of the tests comprising the PACC during the remote assessment
1. Mini mental state examination	1. Immediate face name associative memory test
2. Immediate recall logical memory	
3. Immediate face name associative memory test	2. Immediate recall logical memory
4. *Task-set shifting/response inhibition task*	3. Cued face name associative memory test
5. Delayed recall logical memory test	4. Mini mental state examination
6. Cued face name associative memory test	5. Digit symbol substitution test
7. Cued face name associative memory test	6. Delayed recall logical memory test
8. Digit symbol substitution test	7. Delayed face name associative memory test
9. *Visual short-term memory binding task*	
10. Delayed face name associative memory test	

In Italics two tests which are not part of the PACC and which were not included in the remote assessment

In addition, during the remote assessment, the participants were asked to check and report their internet speed, using a website (http://www.fast.com). At the end of the call they were asked to complete a post-assessment interview to record how they felt about the two assessments compared (in-person and remote). The post-assessment interview comprised of three questions, the first two were ratings on a scale of 1–5 assessing how comfortable the participants felt with the in-person and remote assessments (1 being very uncomfortable and 5 being very comfortable). The third question was open-ended for the participants to give their opinion on the two assessments and if they believed they were comparable. At the end of the assessment, the participant was asked to place in the stamped return envelope the MMSE writing and drawing sheet, the DSST worksheet and the post-assessment interview including a unique ID given to them to be identifiable to the researchers, before they were returned.

A database was created on Excel which included the participant ID, the assessor from the in-person assessment and the assessor for the remote assessment (different assessors were used for the in-person and remote assessments), internet speed of the participant, video software used, date of in-person and remote assessment, completed HEADING lifestyle questionnaire from the in-person assessment, in-person PACC test scores and remote PACC test scores, and the post-assessment interview.

### Data analysis

Descriptive statistics were produced including means and medians and graphical displays of distributions using histograms and scatterplots. Participants were included in the analysis if they had a complete PACC score for both the remote and in-person assessments. Those who participated in both assessments were compared to those who only performed in-person assessments with descriptive statistics, such as mean and medians, Chi-square and t-test and scatterplots. Since the PACC score is bases on the standardized test results of a sample, the PACC score for the in-person assessment was calculated twice, first with all the participants who completed only the in-person assessments, then again for the sample who completed both assessments, the second to be used for the difference measure. The correlation between the two PACC scores was estimated, and the difference between the two PACC scores was calculated. A positive difference implies the remote PACC score is higher than the in-person score, and a negative difference represents a higher in-person score. This difference measure was then used in a multiple linear regression to assess the role of variables potentially associated with a difference in PACC scores. The time elapsed between the two tests was modelled as both a categorical and continuous variable to explore a possible effect of time. Continuous variables (age, time and internet speed) were centered on the mean for the regression analysis. To better interpret the results of the regression, a marginal effect plot was explored on the mean PACC difference in the sample by varying time between assessments. Differences in scores of the individual tests comprising the PACC were also analyzed separately with the same approach. Agreement of the two measures was further assessed with a Bland–Altman plot. All analyses were further run without an identified outlier. All analyses were conducted using STATA statistical package.

## Results

**Fig. 1:**
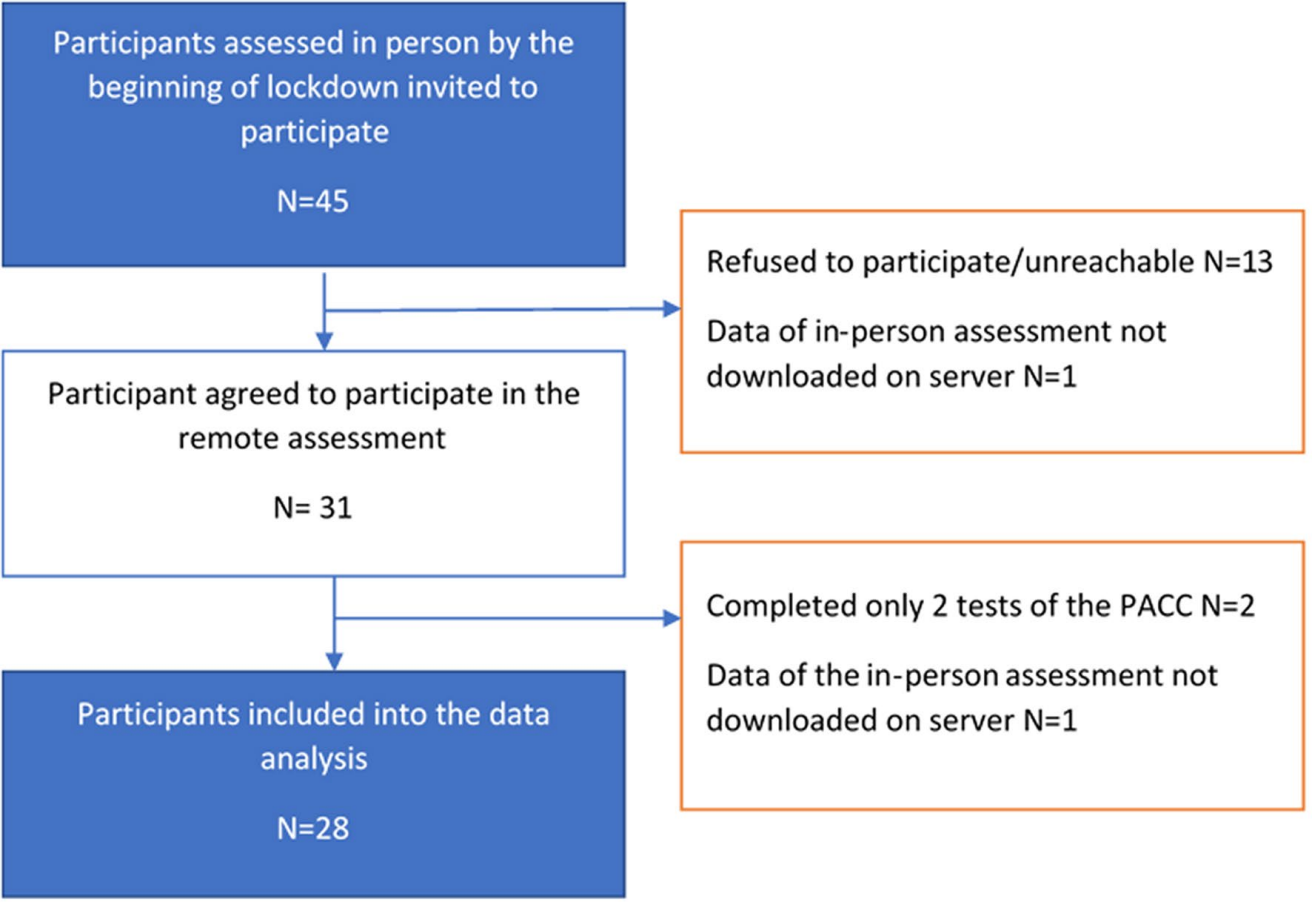
Flowchart of the recruitment into the study and final sample

As of March 13th, 2020, 45 participants had been assessed for the HEADING Study. These 45 participants were invited to take part in the remote assessment; 31(69%) agreed to participate in the remote assessment. At the time of this analysis, there were 30 participants who had completed a virtual PACC assessment and had data available for the in-person assessment (67% of the total). Of the 30 participants in the feasibility study, two participants completed only two of the four tests, with one participant suffering from aphasia and another having recently undergone hand surgery inhibiting their ability to do the DSST and half the MMSE. Therefore, leaving 28 participants (Fig. 1). The median age was 60 years old (IQR 16) (Table 1), with 57% of the participants being educated up to GCSE standard. Only 10% of the participants had ever smoked, and 75% of the participants drank alcohol. A comparison between the original sample, and the participants included in the remote assessment is reported in Table 1. Participants who accepted to be re-assessed were on average younger (*p* = 0.03), more educated (*p* = 0.02) and had a higher PACC score (*p* = 0.05).

**Table 1:** Characteristics of the participants, comparing all the participants who have participated in the heading study with those who participated in both the in-person and remote assessments

	In-person assessment	Remote assessment	Non-respondents	*p*-value*
N^a^	43	28	13	
In-person PACC score, median (IQR)^b^	0.13 (1.08)	0.25 (1.13)	− 0.17 (1.03)^b^	0.05
Time between assessment (days), median (IQR)^c^	–	236.5 (67)	266 (62)	0.30
Age in years, median (IQR)	63 (17)	60 (16)	69 (9)	0.03
Highest educational qualification, *N* (%)^d^				
Primary school	12 (28)	4 (14)	6 (46)	0.02
GCSE	18 (42)	12 (43)	6 (46)	
A level	5 (12)	5 (18)	0	
Undergraduate Degree	7 (16)	6 (21)	1 (8)	
Post graduate degree	1 (2)	1 (4)	0	
Smoke status, *N* (%)				
Current or former smoker	7 (16)	3 (11)	4 (31)	0.11
Never smoked	36 (84)	25 (89)	9 (69)	
Alcohol status, *N* (%)				
Current or former drinker	30 (72)	21 (75)	8 (62)	0.38
Never drink	13 (28)	7 (25)	5 (38)	

^*^p-value for *T* test (continuous) or chi-square test (categorical)

^a^Refer to Fig. 1

^b^One missing, did not have a complete PACC score (missing two tests)

^c^Calculated attributing to participant not re-assessed a time of reassessment from averaging the time of second assessment for the re-assessed

^d^Education regrouped for chi-test: GCSE and below vs. A-level or above

The shortest time between assessments spanned 103 days, while the longest time between assessments was 293 days (3.4 and 9.6 months, respectively). The median duration in days between the in-person and the subsequent remote assessments was 236.5 days (or 7.9 months) (IQR 62.5). When the time between assessments variable was categorized, 5 (17%) participants had less than 149 days (4.9 months) between assessments, three (10%) between 150 and 199 days (4.9–6.5 months), 12 (40%) ranged between 200 and 249 days (6.6–8.2 months) and ten (13%) over 250 days (8.2 months) between assessments. Most of the remote assessments (80%) were performed using applications Skype and Zoom.

**Fig. 2:**
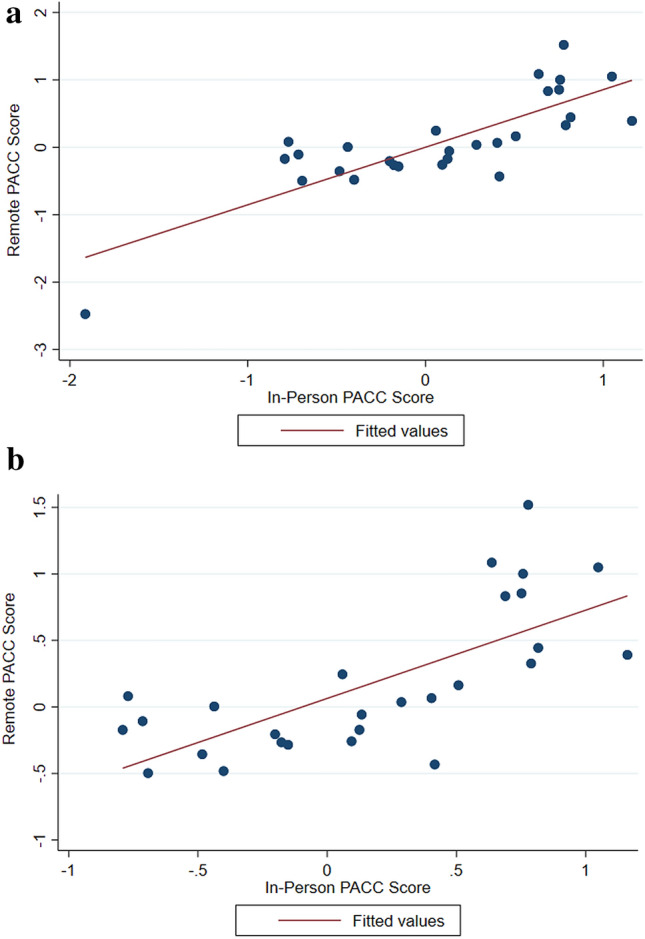
**a** Scatterplot of Individual PACC scores coming from the in-person and remote assessments in the full sample. **b** Scatterplot of individual PACC scores coming from the in-person and remote assessments in the full sample, removing the outlying participant

**Fig. 3:**
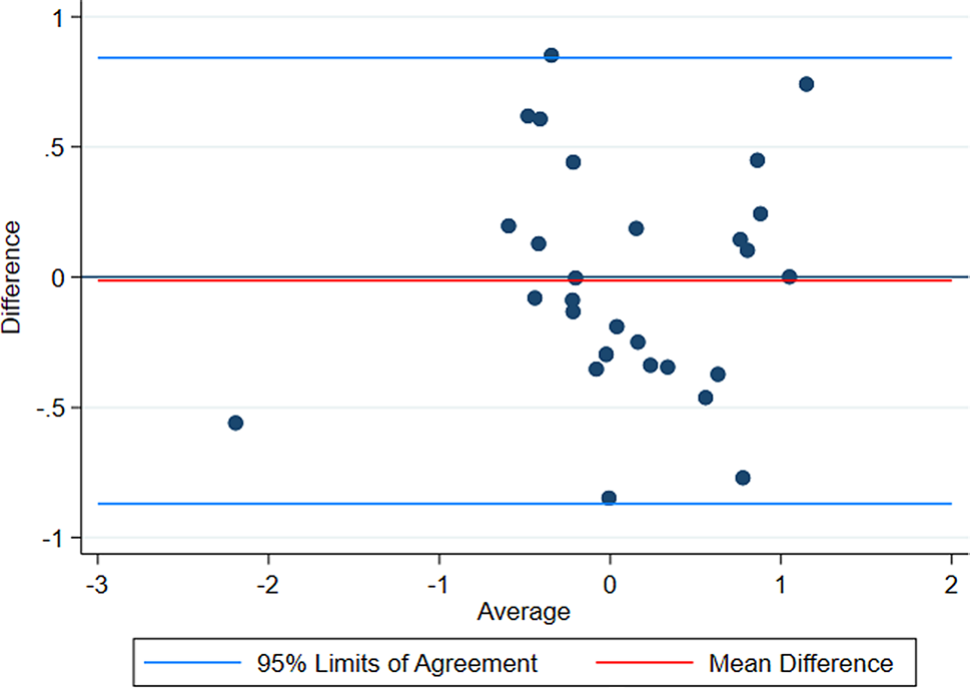
Bland–Altman plot assessing the agreement between the remote PACC and in-person PACC score. The mean difference is − 0.013, with upper limit of agreement being 0.84 and the lower limit of agreement being − 0.87. Difference PACC calculated as remote PACC minus in-person PACC

The PACC scores for the two assessments are plotted in Fig. 2. There was a strong positive correlation between the two assessments for the PACC score, with a Pearson correlation coefficient of 0.82 (95% CI 0.66, 0.98). Summary statistics for the PACC scores and the PACC difference are shown in Table 2. A Bland–Altman plot was further used to assess agreement between the two PACC scores (Fig. 3). This suggests that the difference between in-person and remote assessments are not detected differentially in those with higher or lower scores.

**Table 2:** Summary statistics of the two PACC scores and the PACC difference

	In-person PACC	Remote PACC	PACC difference
Mean	0.097	0.084	− 0.013
Median	0.13	0.02	− 0.042
SD	0.70	0.74	0.44
IQR	1.14	0.68	0.56

PACC difference calculated as the remote PACC minus in-person PACC

**Fig. 4:**
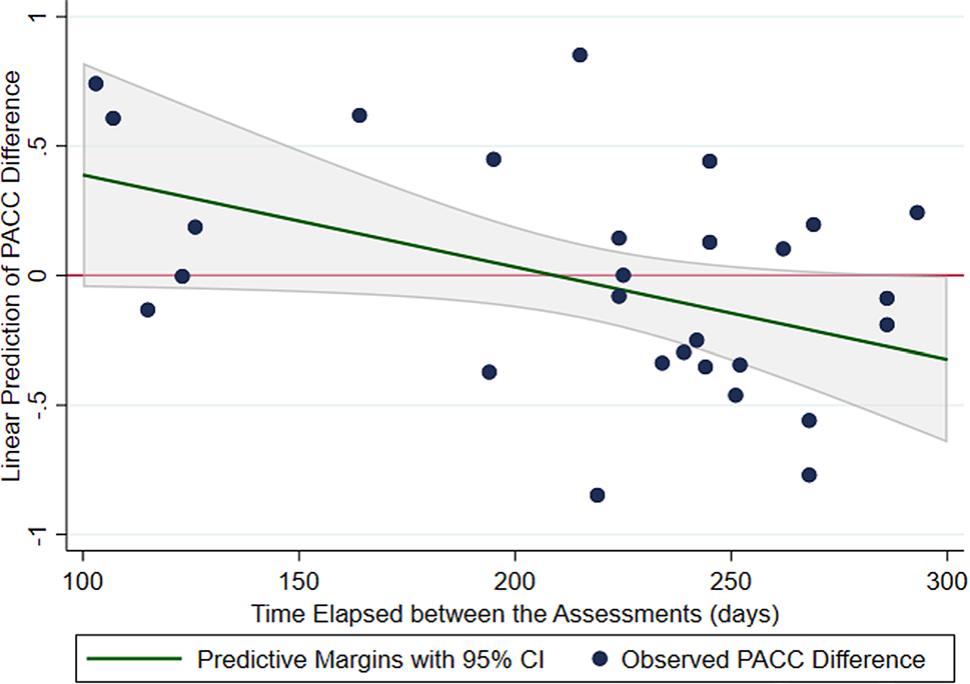
Marginal effects plot of the predicted mean PACC difference of the sample, with varying time between assessments. The plot shows that if less than 200 days passed between assessments, the remote PACC score would be higher than the in-person PACC score (as denoted by a positive PACC difference). Likewise, after 200 days between assessment, the predicted PACC difference suggests the remote PACC score was lower than the in-person PACC score

The multiple linear regression included the age of participants, highest educational qualification, internet speed, in-person and remote assessors, and time between assessments for 27 participants due to a missing value for internet speed (Table 3). The regression produced a constant value of − 0.17 (95% CI − 0.54, 0.19), meaning the expected PACC difference between the in-person PACC was 0.17 points higher than the remote PACC for an ‘average’ person in the sample (specifically, a person aged 61 years, with GCSE education level, assessed by video assessor 1, person assessor 4, with an average time of 218 days between assessments and with an internet speed of 31 mbps). The time between assessments, as a continuous variable, was identified to be associated with the PACC difference. Time between assessment predicted a decrease in PACC difference by − 0.004 points (*β* = − 0.004 95% CI − 0.007, − 0.00008) with increasing time between assessments (in days). This means that the difference in PACC scores between each increasing day will differ on average by 0.004 points. The analysis of marginal effects showed that when the two tests were administered relatively closer in time, the mean difference was positive (the subsequent remote test performance was better), but the difference became negative (in-person performance better) with increasing time difference between the assessments (Fig. 4). When analyzing the individual tests, time between assessments was also identified with the Logical Memory test (data not shown). The analysis run after removing the outlier did not change the results, as shown in Table 3. The responses of the post-assessment questionnaire are displayed in Supplemental Table 1.

**Table 3:** Mean PACC score difference among the factors identified as being associated with the PACC difference after a multiple linear regression

	Model 1^a^: beta coefficient	95% Confidence interval	Model 2^b^: beta coefficient	95% Confidence interval	Model 3^c^: beta coefficient	95% confidence interval
Age	0.0002	− 0.021, 0.21	0.008	− 0.013, 0.028	0.008	− 0.014, 0.03
Internet speed (Mbps)^d^	0.003	− 0.004, 0.01	0.005	− 0.001, 0.011	0.005	− 0.002, 0.011
Time between assessments (days)	− 0.003	− 0.006, − 0.0006	− 0.004	− 0.007, − 0.00008	− 0.004	− 0.007, 0.0001
Educational qualification						
GCSE	*Ref*	*Ref*	*Ref*	*Ref*	*Ref*	*Ref*
Primary school	0.28	− 0.36, 0.91	0.43	− 0.10, 0.95	0.42	− 0.14, 0.98
A level	0.045	− 0.50, 0.59	− 0.005	− 0.46, 0.45	− 0.009	− 0.50, 0.48
Undergraduate	0.037	− 0.46, 0.53	0.16	− 0.23, 0.55	0.16	− 0.27, 0.58
Post-graduate	− 0.24	− 1.30, 0.83	− 0.67	− 1.58, 0.23	− 0.68	− 1.62, 0.27
Video assessor						
1	*Ref*	*Ref*	*Ref*	*Ref*	*Ref*	*Ref*
2	− 0.22	− 0.67, 0.22	− 0.17	− 0.60, 0.26	− 0.17	− 0.62, 0.28
3	0.18	− 0.27, 0.63	0.42	− 0.014, 0.85	0.42	− 0.034, 0.86
Person assessor						
4	*Ref*	*Ref*	*Ref*	*Ref*	*Ref*	*Ref*
5	− 0.12	− 0.49, 0.25	0.041	− 0.38, 0.46	0.039	− 0.41, 0.48
Constant	− 0.30	− 0.21, 0.15	− 0.17	− 0.54, 0.19	− 0.17	− 0.56, 0.23

Time between assessments, age and internet speed centered on the mean

^a^Adjusted for age only, *N* = 28

^b^Adjusted for all variables in the table, *N* = 27

^c^Adjusted for all variables in table, excluding outlier, *N* = 26

^d^One missing value

*Mbps* megabits per second

Overall, the participants reacted well to the remote assessment, with 22 (78%) participants responding that they felt extremely comfortable performing the remote assessment on the post-assessment interview, with 3 participants scoring the remote assessment worse than the in-person assessment (Supplemental Table 1). Participants mentioned that although there are more problems with remote assessment (interrupted internet signal, confusion in setting up) the testing process felt equal to that already performed in-person.

## Discussion

This study provides evidence of the feasibility of administering the tests comprising the PACC score via videoconferencing: administering the tests sending the participants some material via the post, in advance was shown to be feasible. The differences between the in-person and remote-administered PACC were overall very small. There weren’t systematic differences between the two PACC scores, arguing against a potential bias introduced by the remote assessment: those who performed worse presented similar in-person/remote differences compared to those who performed best.

The only variable which predicted the PACC score differences was the time between assessments; its association with the PACC difference being very small. The marginal effect plot showed a tendency of better performance at the remote assessment, on average, when the time difference between the two was shorter, but this association reversed as the time between assessments increased. This could be interpreted as a potential learning effect—given that all the participants had already undergone the face-to-face assessment prior to the remote assessment- which wears off over time [15, 16]. A learning effect involves increases in repeated test scores due to factors such as memory for specific test items, learned strategies for problem solving or general experience and comfort with testing. This learning effect was observed despite the average time between tests was 7.2 months in this study, which is longer than the two to three months other studies have used when considering learning effects [7, 10] suggesting that the learning effect in substantially cognitively intact people could be longer than previously recognized. This difference between the two measures could also be interpreted as participants being in a more comfortable setting in their home performing better [17].

The fact that the learning effect wears off over time is predictable, less easy to interpret is a tendency toward a reverse effect (remote assessment worse than in person). One possible interpretation is that it is a detection of very subtle cognitive decline over time among the participants. The BRAIN study has shown that PACC scores decrease with age among retired rugby players (manuscript under review) [5]. Given the long test–retest interval between the two assessments, this could be a possibility. The PACC was originally established to measure cognitive decline over time, being administered every 6 months over the course of 36 months. However, an average of seven months between the assessments may not be long enough to detect a change in cognitive function, as seen by Donohue et al., where the earliest detectable change in PACC scores was at 12 months [4]. Finally, it is not possible to rule out completely that different assessors for the in-person and remote assessments might have had an effect on the scoring, although this was also adjusted for in the analysis.

Importantly, the effect of time on the scoring difference is not relevant for the HEADING and many other epidemiological studies which use the remote PACC assessment solely in person, or remotely. The high correlation between the two sets of tests and the absence of a clear bias affecting disproportionally people performing less well, suggest that this is a valid method to be used in epidemiological studies on a given population with high computer literacy and cognitively normal.

Participants accepted well the remote cognitive testing finding it comfortable to be assessed remotely, nonetheless all of them had already undergone the assessment, so they knew what to expect. It remains to be explored if participants assessed remotely only would self-rate the assessment as comfortable as well.

### Limitations

The response rate to reassessment was not ideal (65%), introducing a potential for selection bias, as those who did not participant were different to those who did. For example, the sample who agreed to participate declared to be more adept in using technology and have videoconferencing devices and internet connection available to them. Of those who declined to participate, two mentioned they either did not have a device for videoconferencing, or they had no internet connection. Studies found that participants who are less computer literate have increased computer anxiety, which could affect scores of computer tests [9, 11]. Therefore, this makes our results not immediately generalizable to a less technologically confident population as the same correlation may not be found. Likewise, participants who did not agree to participate had a longer interval of time between assessments, with a median of 266 days (q25, q75: 206, 268). It is unlikely that cognitive status changed over the course between the in-person assessment and the retest, however this is a possibility. The median PACC of the in-person assessments was lower in those who did not agree to participate in the remote assessment, compared to those who did (*p* = 0.05). This would potentially introduce a bias towards a more cognitively able population in the results.

### Contextualization of results

The present results are in line with previous studies comparing face-to-face and virtual assessments [7–13, 18–20]. Telemedicine is a growing field, becoming more relevant particularly with regards to assessing cognitive function, as it has an advantage of reaching more participants and reducing the burden of lengthy travelling, cutting time, cost and making the participant feel more comfortable in their own home [7, 11, 19, 20]. Moreover, telemedicine can be used to reduce in-person contact to abide by recent government guidelines by increasing the possibility of social distancing as well as the security of being able to reach at risk participants during these times. In this study, the use of videoconferencing was chosen over telephone assessment because the PACC assessment involved the need to see the participant performing tasks, as well as sharing the screen for the Face-Name Association Task. Nonetheless, videoconferencing can be seen as more insightful because it captures non-verbal cues that cannot be done in telephone interviews such as facial expressions and attentiveness [7].

Conversely, compared to face-to-face assessments, remote assessments have some disadvantages, such as loss of attention due to surroundings as well as the potential for participants writing down answers or looking at a calendar. For instance, this study noted one participant being distracted during the video call by their surroundings at home, while another participant received a phone call during the digit symbol substitution test. The analysis accounted for internet speed to adjust for potential connection problems that could have interfered with the assessment. Furthermore, the analysis took into account the software used for the videoconferencing, however, what was not taken into account was the device used, as the visual cues, particularly with the FNAME, could be altered on a smartphone compared to on a computer, as the stimuli would be smaller. Stillerova et al. [9] assessed remote testing with different software and different devices and found no difference among modes used. The marking of the overall scores can also be adjusted for video assessment, such as Timpano et al. [8] lowering the cut off for the virtual MMSE, to account for poor internet speed and other factors that could influence the assessment. Other potential problems, such as writing down questions and changing answers, were addressed by ensuring that participants showed their responses for the MMSE writing and drawing tasks and for the digit symbol substitution test. Besides the disadvantages that may arise from remote assessments, this study also had a limitation of a small sample size, thus introducing variability in the results and reducing statistical power, limiting the ability for clear interpretation of the results. This reduction in power is also denoted by the large confidence intervals seen in the correlation coefficient, preventing the reliability of the results to be generalized to a broader audience. Further non-linear trends of the effect of time could therefore not be explored given the small sample size. A sensitivity analysis removing the outlier did not affect the results.

While the HEADING study recruits participants with high cognitive ability (median MMSE 29 (28, 30)), making the remote assessment feasible, the results cannot be generalized to mild or cognitively impaired populations. The main problem with evaluating individuals with more severe cognitive impairments remotely is usually due to technology. Researchers have shown that remote testing can be achieved on participants with Alzheimer’s, dementia and movement disorders [11, 12, 18, 20]. However, in these instances, remote assessments have been done in clinical settings, with a trained nurse assisting in the delivery of the assessment. This is beyond the scope of this feasibility study, as we would have had to train care givers to set up an appropriate environment remotely and to supervise the assessments in a thorough manner. Other problems of evaluating those with more advanced symptoms has been found that they usually have trouble maintaining and sustaining attentions, and this can be overcome by keeping the remote evaluations brief [7].

The factors mentioned above (cognitive function of the participants, time between assessment and learning effects) all affect the test–retest reliability of the presented results. The correlation found accounts for 82% of the variance (95% CI 0.66, 0.98), however, should be interpreted with caution given the small sample size and characteristics of the sample. With this said, other validation studies of the PACC have found high test/retest reliability (*r* = 0.94, *p* < 0.001) in their sample [21]. Looking at the individual tests included in the PACC, tests involving attention, processing speed and working memory tend to be more susceptible to longer test–retest intervals, while tests involving episodic memory are more susceptible to learning effects [22], with the MMSE also showing poor reliability and ceiling effects [23]. However, a meta-analysis found that comparing reliabilities across studies is difficult given the study population, length of test interval, and tests used [24]. Many other methodological issues arise when measuring cognitive function, and potential decline over time, in epidemiological studies, and are stated elsewhere [25].

In conclusion, when considering limitations, these data support the feasibility of conducting the PACC assessment remotely. This feasibility study showed how the use of videoconferencing software that are all freely available to download, are easy to use by study participants and fit for the scope. This is an advantage in epidemiological studies, as it can reduce cost and time involved with in-person assessments as well as adapt to uncertain circumstances when face-to-face assessments are not possible. To further understand whether remote testing can replace in-person assessments, a larger sample size and more adequate study design, will need to be used.

## Supplementary Information

Below is the link to the electronic supplementary material.

Supplementary file1 (DOCX 39 KB)

## Data Availability

Dr. Gallo had full access to all of the data in the study and takes responsibility for the integrity of the data and the accuracy of the data analysis. She declares that this manuscript is honest, accurate, and transparent account of the study being reported; that no important aspects of the study have been omitted. All co-authors had full access to the data and can take responsibility for the integrity of the data and the accuracy of the data analysis.
