# Supplementary material for: Feasibility study of assessing the Preclinical Alzheimer Cognitive Composite (PACC) score via videoconferencing
Source: J Neurol. 2021 Jan 26;268(6):2228–37. doi: 10.1007/s00415-021-10403-1 (PMC7836343; doi:10.1007/s00415-021-10403-1)
Supplement: Supplementary file 1 — Supplementary file1 (DOCX 39 KB) [file 415_2021_10403_MOESM1_ESM.docx]

Supplementary Table 1: Participants rating on the post assessment questionnaire, rating their experience between the two assessments, with 1 meaning “extremely uncomfortable” and 5 meaning “very comfortable”

|  | In- Person Rating | |
| --- | --- | --- |
|  | **4** | **5** |
| Remote Rating | **N (%)** | **N (%)** |
| 3 | 1 (20) | - |
| 4 | 3 (60) | 2 (8.7) |
| 5 | 1 (20) | 21 (96.3) |
